# Ten-year trajectory of coronary artery calcification and risk of cardiovascular outcomes: the Multi-Ethnic Study of Atherosclerosis

**DOI:** 10.3389/fcvm.2024.1406216

**Published:** 2024-07-10

**Authors:** Changming Xie, Dongling Luo, Guodu Liu, Jie Chen, Hui Huang

**Affiliations:** ^1^ Department of Cardiology, The Eighth Affiliated Hospital, Joint Laboratory of Guangdong-Hong Kong-Macao Universities for Nutritional Metabolism and Precise Prevention and Control of Major Chronic Diseases, Sun Yat-sen University, Shenzhen, China; ^2^ Department of Radiation Oncology, Sun Yat-sen Memorial Hospital, Sun Yat-sen University, Guangzhou, China

**Keywords:** coronary artery calcification, trajectory, cardiovascular outcomes, coronary heart disease, risk management, senescence

## Abstract

**Background:**

Whether and how coronary artery calcium (CAC) progress contributes to cardiovascular outcomes has not been fully elucidated. The aim of this study was to identify different patterns of CAC change and evaluate the associations with different cardiovascular outcomes.

**Methods:**

Data from the Multi-Ethnic Study of Atherosclerosis study were analyzed. Participants with at least three CT measurements were included. The main study outcome is hard cardiovascular disease (CVD). CAC scores were determined as phantom-adjusted Agatston scores. A group-based trajectory model was used to identify latent groups and estimated the hazard ratios (HR) and 95% confidence intervals (CI) using Cox proportional regression models.

**Results:**

A total of 3,616 participants were finally enrolled [mean age 60.55 (SD 9.54) years, 47.76% men and 39.30% Caucasian]. Four distinct trajectories in CAC were identified: class 1, low-stable (24.17%); class 2, low-increasing (27.60%); class 3, moderate-increasing (30.56%); and class 4, elevated-increasing (17.67%). During 13.58 (SD 2.25) years of follow-up, 291 cases of hard CVD occurred. The event rates of hard CVD per 1,000 person-years were 2.23 (95% CI 1.53–3.25), 4.60 (95% CI 3.60–5.89), 7.67 (95% CI 6.38–9.21) and 10.37 (95% CI 8.41–12.80) for classes 1–4, respectively. Compared to participants assigned to class 1, the full-adjusted HRs of hard CVD for classes 2–4 were 2.10 (95% CI 1.33–3.01), 3.17 (95% CI 2.07–4.87), and 4.30 (95% CI 2.73–6.78), respectively. The graded positive associations with hard CVD were consistently observed in subgroups of age, sex, and race, with the presence or absence of hypertension or diabetes. By analyzing potential risk factors for distinctive CAC trajectories, risk factors for the onset and progression of CAC could possibly differ: age, male sex, history of hypertension, and diabetes are consistently associated with the low-, moderate-, and elevated-increasing trajectories. However, Caucasian race, cigarette smoking, and a higher body mass index was related only to risk of progression but not to incident CAC.

**Conclusion:**

In this multi-ethnic population-based cohort, four unique trajectories in CAC change over a 10-year span were identified. These findings signal an underlying high-risk population and may inspire future studies on risk management.

## Introduction

Coronary artery calcification is an important facet of coronary heart disease (CHD) and has been established as a strong risk-predictor for future cardiac events, which could be easily diagnosed using imagological examination (1–4). However, emerging data have suggested vascular calcification is not a passive process as it was believed; instead, it is an organized and active pathogenic process (5–7). Thus, a longitudinal analysis of coronary artery calcium (CAC) seems essential (8, 9).

Over the past decade, an increasing number of studies have suggested a link between CAC progression and subsequent events (10, 11). However, at least 10 different algorithms have been used to report CAC progression, based on two CT measurements within a short-period of observation duration (12–14). Some researchers estimate the absolute change or percent change to quantify the progression rate (10, 11, 15). Others create categorical progression by artificially setting a grouping range (16–18). This could be heavily influenced by outliers or measurement errors and depends heavily on the chosen thresholds, making it difficult to draw conclusions about actual population patterns. Comparing different definitions of CAC progression, Paixao et al. pointed out that the various definitions can result in divergent subject classification in up to 30% (14). Furthermore, the choice of scale for the analysis of CAC progression may lead to incongruent associations with cardiovascular disease (CVD). In a recent analysis of the Multi-Ethnic Study of Atherosclerosis (MESA) study, CAC progression is a risk marker for future hard and total CHD events (11). This was later challenged by other studies, such as Lehmann et al. stating that what matters is the most recent CAC value and not its progression rate (13). Thus, how the progress of CAC contributes to subsequent events has not been fully elucidated.

To overcome these issues, group-based trajectory models (GBTM) were applied in this study. This approach allows the conceptualization of the growth and development of CAC change and identification of clusters of individuals following similar patterns of change over time (19).

Using at least three repetitive CT measurements over a 10-year span, the aim of the present study was to (1) identify different patterns of CAC change; (2) evaluate the associations of CAC trajectories with different cardiovascular outcomes; and (3) try to investigate risk factors associated with distinct patterns of CAC trajectories.

## Methods

### Setting and participants

The design of the MESA study has been described in detail previously (20). In brief, a total of 6,814 participants aged 45–84 years and free of CVD at baseline visit (exam 1, 2000–2002) were initially recruited from six field centers. Follow-up examinations were conducted during 2002–2003 (exam 2), 2004–2005 (exam 3), 2005–2007 (exam 4), and 2010–2012 (exam 5). Per protocol, CAC was measured for all participants at study entry and repeated during the follow-up (20). In our analyses, we only included participants with at least three CT measurements, finally enrolling a total of 3,616 individuals. The studies involving human participants were reviewed and approved by the Ethics Committee of MESA. The patients/participants provided their written informed consent to participate in this study.

### Coronary artery calcium assessment

Methodology of CT scans and CAC measurements have been well-described in previous reports (21, 22). Briefly, either electron-beam or multi-detector CT (three sites) was used to scan these participants (11, 23). Two consecutive measurements were carried out and the results were read by a trained professor at a centralized reading center (Los Angeles Biomedical Research Institute, Torrance, CA, USA) (11). CAC scores were determined as phantom-adjusted Agatston scores by averaging the results from the two scans. Since the CAC scores were non-normally distributed data, they were converted to logarithmic values [log(CAC + 1)] before analysis (8, 17, 24).

### Measurements of other covariates

Information on demographic characteristics, lifestyle factors, and past histories was obtained through standardized questionnaires or measured by health interviewers. Blood pressures were measured using an oscillometric method. Body mass index (BMI) was calculated by weight (kg) divided by square of height (m^2^). History of hypertension was defined as systolic blood pressure (BP) ≥140 mmHg and diastolic BP ≥90 mmHg, self-reported hypertension, or using anti-hypertensive medications. History of diabetes mellitus was defined as self-reported diabetes, a fasting serum glucose ≥126 mg/dl or use of anti-diabetic medications. Fasting serum glucose, creatinine, total cholesterol, high-density (HDL-C) or low-density lipoprotein cholesterol (LDL-C), and triglycerides were measured as previously described (20). Daily consumptions of different nutrients were quantified by data from a 120-item food frequency questionnaire during the previous year. Other available variables of interests used in the current study included age, sex, level of education, marital status, smoking status, alcoholic use, family income, physical activity, total energy consumption, dietary calcium, phosphate, and vitamin D.

### Outcome ascertainment

Clinical events were ascertained by telephone interviews every 9–12 months, with all events adjudicated by an independent MESA committee (20). Participants were followed from baseline exam 1 until they experienced events of interest, were lost to follow-up, or until 31 December 2015. The main outcome in this study is hard CVD, including a composite of myocardial infarction (MI), resuscitated cardiac arrest, stroke (not transient ischemic attack), CHD death, and stroke death, which is defined and has been approved by the MESA Steering Committee. All CHDs (including MI, resuscitated cardiac arrest, definite angina, probable angina, and CHD death), chronic heart failure (CHF), and stroke were examined as secondary outcomes.

### Statistics analysis

We used a GBTM to identify latent groups in participants’ CAC trajectories with the user-written program “traj” for STATA (25). Repeated CAC measurements were fitted as a mixture of several latent trajectories in a censor normal model with a polynomial function of age (e.g., linear, quadratic). The adequacy of the final model was evaluated using a low Bayesian information criterion (BIC) value and a probability of belonging higher than 0.70 (26, 27). Details of the selection process are presented in the Supplementary Materials.

Descriptive statistics are presented as means ± standard deviations (SD) for continuous variables and proportions for categorical variables according to the exposure trajectories.

Cox proportional hazards regression was used to examine the association of identified CAC trajectories with the studied outcomes, taking the low-stable group as reference. We used three models with increasing degrees of adjustment to assess the associated risks: (1) crude model (non-adjusted); (2) simple-adjusted model (adjusted for age, sex, race, alcoholic use, level of education, marital status, family income); and (3) full-adjusted model (adjusted for age, sex, race, alcoholic use, level of education, marital status, family income, body mass index, history of hypertension, diabetes, smoking status, physical activity, total energy consumption, dietary calcium, vitamin D and phosphate, LDL-C, HDL-C, total cholesterol, triglycerides, glucose, creatinine, systolic blood pressure, diastolic blood pressure, anti-hypertensive medication, anti-diabetic medication, and lipid-lowering medication). Results were reported as hazard ratios (HR) and 95% confidence intervals (95% CI). The proportional hazards assumption was checked by plotting the log[-log(survival)] versus log (survival time). We did not find evidence suggesting potential violation of these results.

Missing values on all sociodemographic covariates were handled by the Markov Chain Monte Carlo multiple imputation method and the results from 10 multiple imputation cycles were combined to draw a final output. The variables entered in the imputation method were potential confounders with missing values. The proportion of missing values was presented in Supplementary Table S1.

To mitigate potential bias, we repeated the main analysis after excluding participants with missing data on baseline covariates and performed a series of subgroup analyses. The association between different CAC trajectories and hard CVD was examined by subgroups of age (≤65 or >65 years), sex (male or female), race (Caucasian or non-Caucasian), hypertension (yes or no), and diabetes (yes or no).

In addition, univariate multinomial logistic regression models were used to identify potential risk factors for the identified trajectories. A change of the effect estimate >1% and a *p*-value <0.05 were further involved in the multivariate multinomial logistic regression models. To further evaluate the characteristics of traditional cardiovascular risk factors for the identified CAC trajectories, we estimated the dynamic change of systolic blood pressure, diastolic blood pressure, blood glucose, HDL-C, LDL-C, and BMI over time within each trajectory group. Second-order fractional polynomial models were fitted using the mfp and fracpred commands in STATA.

All data were analyzed using STATA version 15.1 (StataCorp/SE, College Station, TX, USA). All statistical tests were two-sided and the significance level was set at 0.05.

## Results

### Baseline characteristics of the study population

**Figure 1:**
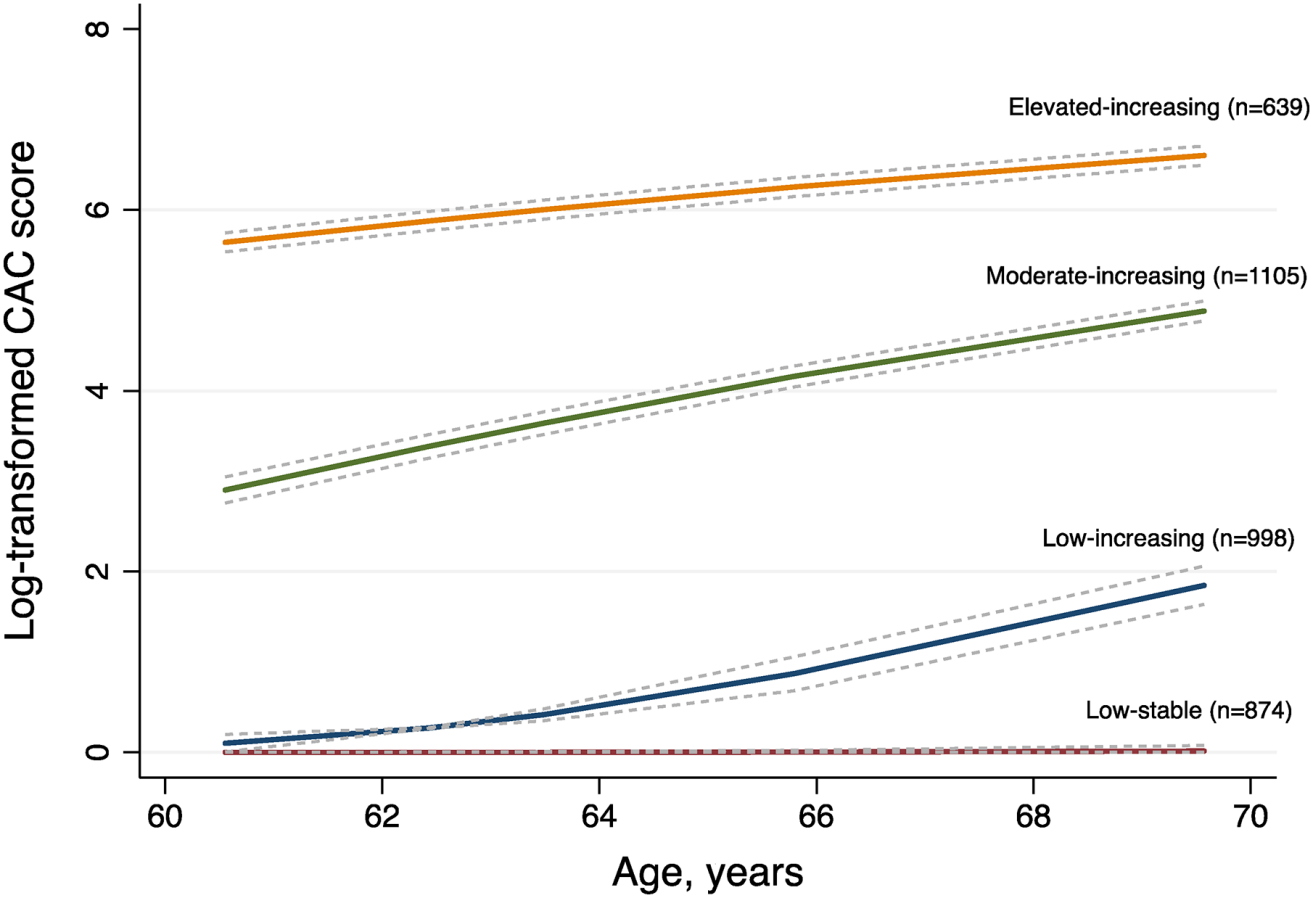
Ten-year trajectories in coronary artery calcium (CAC) in the MESA study. Solid lines represent the trajectory class identified for the estimated pattern of CAC scores by age, with the corresponding dashed lines representing 95% confidence intervals. The data in parentheses represent the number of participants in each class.

**Figure 2:**
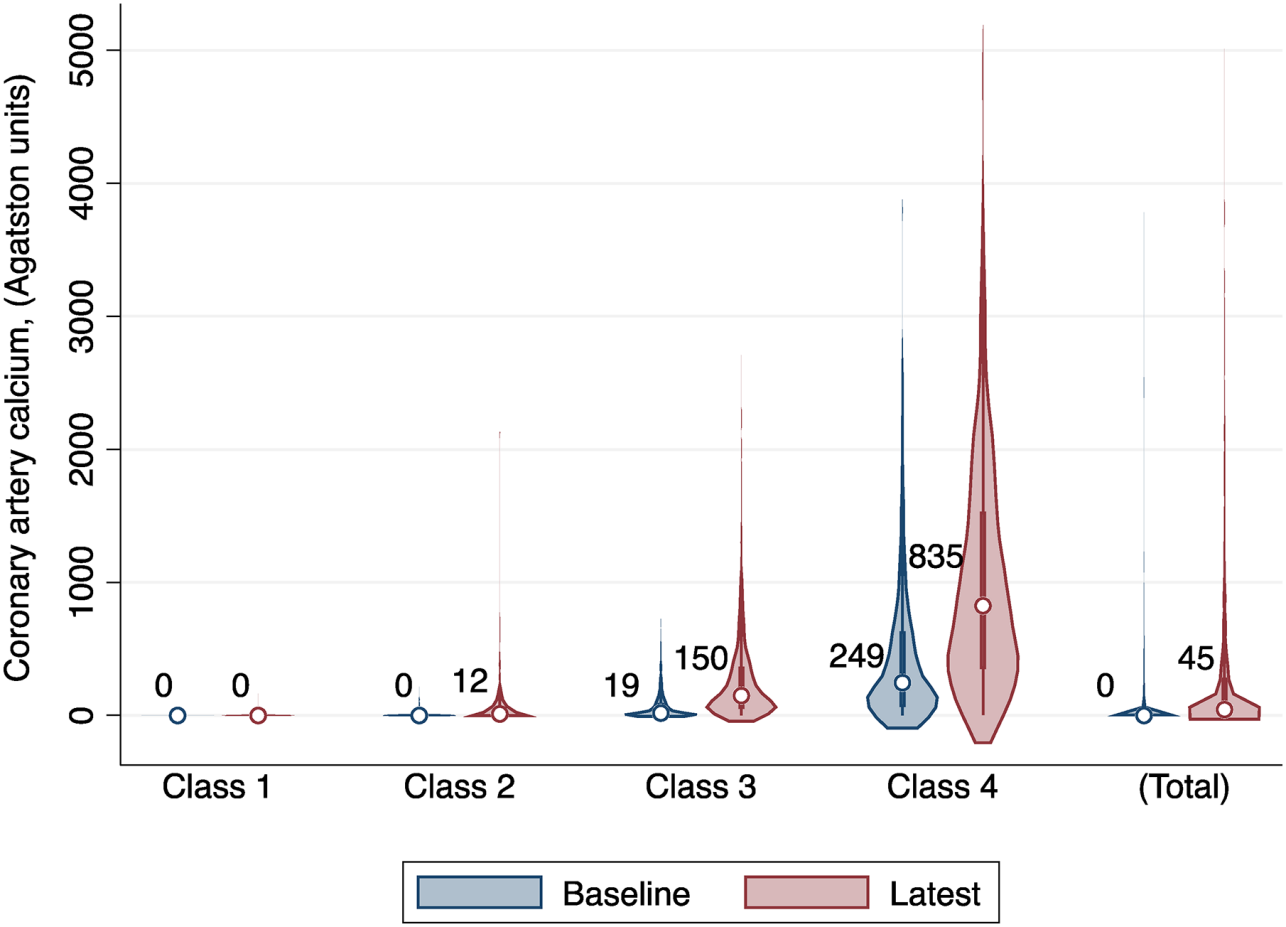
The distribution of baseline and latest coronary artery calcium (CAC) scores across distinct CAC trajectory groups. Median baseline and latest CAC scores are presented under each class. Class I: low-stable group; class 2: low-increasing group; class 3, moderate-increasing group; class 4: elevated-increasing group.

We enrolled a total of 3,616 participants in this study, among whom 78.29% (2,831/3,616) had three CT measurements and 21.71% (785/3,616) had four. The mean age of study participants at enrollment was 60.55 years (SD 9.54), 47.76% were men, and 39.30% were Caucasian. We compared the BIC values and average posterior probabilities (AVPP) in models with 1–6 classes with linear or quadratic functions. The final models were selected based on a low BIC and high probabilities of belonging. Finally, four distinct trajectories in CAC were identified (Figure 1): 24.17% (*n* = 874) of the participants were assigned in the low-stable class (class 1); 27.60% (*n* = 998) in the low-increasing class (class 2); 30.56% (*n* = 1,105) in the moderate-increasing class (class 3), and 17.67% (*n* = 639) in the elevated-increasing class (class 4). The mean AVPPs were 0.82 (SD 0.20), 0.78 (SD 0.22), 0.88 (SD 0.14), and 0.90 (SD 0.15) for those assigned in classes 1–4, respectively. The distribution of baseline and latest CAC scores across groups is depicted in the violin plot (Figure 2). Baseline characteristics, including sociodemographic information, health behaviors, and biomarkers according to different CAC trajectory groups, are summarized in Table 1.

**Table 1:** Baseline characteristics of the study population according to the distinct coronary artery calcium trajectories.

	Total (*n* = 3,616)	Low-stable (*n* = 874)	Low-increasing (*n* = 998)	Moderate-increasing (*n* = 1,105)	Elevated-increasing (*n* = 639)	*p*-value
Age, years	60.55 (9.54)	61.73 (8.46)	59.58 (10.59)	60.98 (9.41)	59.73 (9.22)	0.102
Gender, male	1,727 (47.76%)	271 (31.01%)	403 (40.38%)	600 (54.30%)	453 (70.89%)	<0.001
Race						<0.001
Caucasian	1,421 (39.30%)	290 (33.18%)	348 (34.87%)	457 (41.36%)	326 (51.02%)	
Non-Caucasian	2,195 (60.70%)	584 (66.82%)	650 (65.13%)	648 (58.64%)	313 (48.98%)	
Education level						<0.001
Below high school	516 (14.29%)	159 (18.23%)	137 (13.74%)	154 (13.95%)	66 (10.33%)	
High school or some college	1,257 (34.80%)	284 (32.57%)	353 (35.41%)	394 (35.69%)	226 (35.37%)	
College and above	1,839 (50.91%)	429 (49.20%)	507 (50.85%)	556 (50.36%)	347 (54.30%)	
Marital status						<0.001
Married	2,288 (63.75%)	523 (60.39%)	635 (64.01%)	689 (62.81%)	441 (69.56%)	
Not married	1,301 (36.25%)	343 (39.61%)	357 (35.99%)	408 (37.19%)	193 (30.44%)	
Alcoholic use	2,939 (81.37%)	657 (75.34%)	800 (80.24%)	923 (83.61%)	559 (87.48%)	
Smoking status						<0.001
Never-smoker	552 (55.37%)	517 (59.29%)	530 (48.01%)	257 (40.22%)	1,856 (51.38%)	
Ex-smoker	328 (32.90%)	270 (30.96%)	449 (40.67%)	279 (43.66%)	1,326 (36.71%)	
Current smoker	117 (11.74%)	85 (9.75%)	125 (11.32%)	103 (16.12%)	430 (11.90%)	
History of diabetes	874 (24.23%)	159 (18.28%)	231 (23.19%)	284 (25.75%)	200 (31.35%)	<0.001
History of hypertension	1,527 (42.23%)	334 (38.22%)	394 (39.48%)	484 (43.80%)	315 (49.30%)	<0.001
Anti-hypertensive medication	1,282 (35.46%)	260 (29.75%)	338 (33.87%)	403 (36.50%)	281 (43.97%)	<0.001
Lipid-lowering medications	592 (16.38%)	104 (11.90%)	141 (14.13%)	203 (18.39%)	144 (22.54%)	<0.001
Anti-diabetic medication	313 (8.66%)	45 (5.15%)	79 (7.93%)	88 (7.96%)	101 (15.83%)	<0.001
Systolic BP, mmHg	132.72 (18.14)	131.25 (18.59)	131.48 (18.51)	133.69 (17.27)	135.02 (18.10)	<0.001
Diastolic BP, mmHg	75.11 (9.92)	73.73 (10.33)	74.19 (10.13)	75.78 (9.35)	77.25 (9.53)	<0.001
Body mass index, kg/m^2^	28.40 (5.28)	27.85 (5.31)	27.98 (5.31)	28.75 (5.25)	29.22 (5.08)	<0.001
Glucose, mg/dl	95.56 (26.38)	91.54 (18.14)	94.06 (24.73)	96.64 (27.88)	101.48 (33.57)	<0.001
LDL cholesterol, mg/dl	117.70 (30.82)	114.70 (29.39)	117.19 (29.65)	119.20 (31.44)	120.01 (33.09)	<0.001
HDL cholesterol, mg/dl	50.82 (14.70)	55.13 (15.62)	51.98 (14.81)	48.71 (13.55)	46.77 (13.37)	<0.001
Total cholesterol, mg/dl	194.33 (34.73)	194.01 (33.90)	193.56 (34.47)	194.53 (34.50)	195.64 (36.64)	<0.001
Triglycerides, mg/dl	130.30 (80.96)	122.35 (87.53)	121.41 (68.28)	135.20 (79.04)	146.51 (89.69)	<0.001
Creatinine, mg/dl	0.95 (0.21)	0.92 (0.18)	0.93 (0.21)	0.96 (0.22)	0.99 (0.25)	<0.001
Dietary energy, kcal	1,538.05 (781.66)	1,486.05 (754.08)	1,529.92 (794.14)	1,523.24 (782.93)	1,649.44 (788.62)	<0.001
Dietary vitamin D, μg	4.42 (3.73)	4.41 (3.79)	4.33 (3.63)	4.41 (3.66)	4.58 (3.91)	0.084
Dietary calcium, mg	717.49 (514.32)	718.04 (521.17)	707.03 (500.05)	717.09 (507.41)	734.09 (539.30)	<0.001
Dietary phosphate, mg	1,036.47 (571.58)	1,011.47 (563.61)	1,026.26 (568.69)	1,034.86 (569.81)	1,090.53 (588.08)	<0.001
Physical activity, MET-min/week	3,469.04 (3,986.64)	3,087.39 (3,605.51)	3,451.34 (3,995.60)	3,455.53 (4,100.52)	4,041.39 (4,206.03)	<0.001

In general, participants who maintained a low-stable CAC score were more likely to be female and non-Caucasian, less likely to drink or smoke, and had a lower proportion of hypertension and diabetes. They were less educated but had better metabolically favorable profiles (lower BMI, lower fasting glucose, lower blood pressure, lower LDL-C, and higher HDL-C).

### CAC trajectories and the studied outcomes

During a mean follow-up of 13.58 years (SD 2.25), hard CVD occurred in 291 of the study participants. Overall, there was a graded positive association between CAC trajectories and cardiovascular risks. The event rates of hard CVD per 1,000 person-years by the exposed trajectories (from class 1 to class 4) were 2.23 (95% CI 1.53–3.25), 4.60 (95% CI 3.60–5.89), 7.67 (95% CI 6.38–9.21), and 10.37 (95% CI 8.41–12.80). Compared to participants assigned to class 1 (low-stable), the non-adjusted HRs of hard CVD for class 2 (low-increasing), class 3 (moderate-increasing), and class 4 (elevated-increasing) were 2.08 (95% CI 1.32–3.26), 3.48 (95% CI 2.29–5.29), and 4.74 (95% CI 3.07–7.29), respectively. The risk estimates persisted after simple adjustments with age, sex, race, use of alcohol, level of education, marital status, and family income. Further inclusion of the cardiovascular risk factors in the full-adjusted model did not notably alter the HR of hard CVD with CAC trajectories (HR 2.10, 95% CI 1.33–3.01 for class 2; HR 3.17, 95% CI 2.07–4.87 for class 3; HR 4.30, 95% CI 2.73–6.78 for class 4). Exclusion of participants with missing data on baseline covariates yielded similar results (Supplementary Table S2). Moreover, the graded associations between CAC trajectories and hard CVD were consistently observed in subgroups of age, sex, and race, with the presence or absence of hypertension or diabetes (Supplementary Table S2).

**Figure 3:**
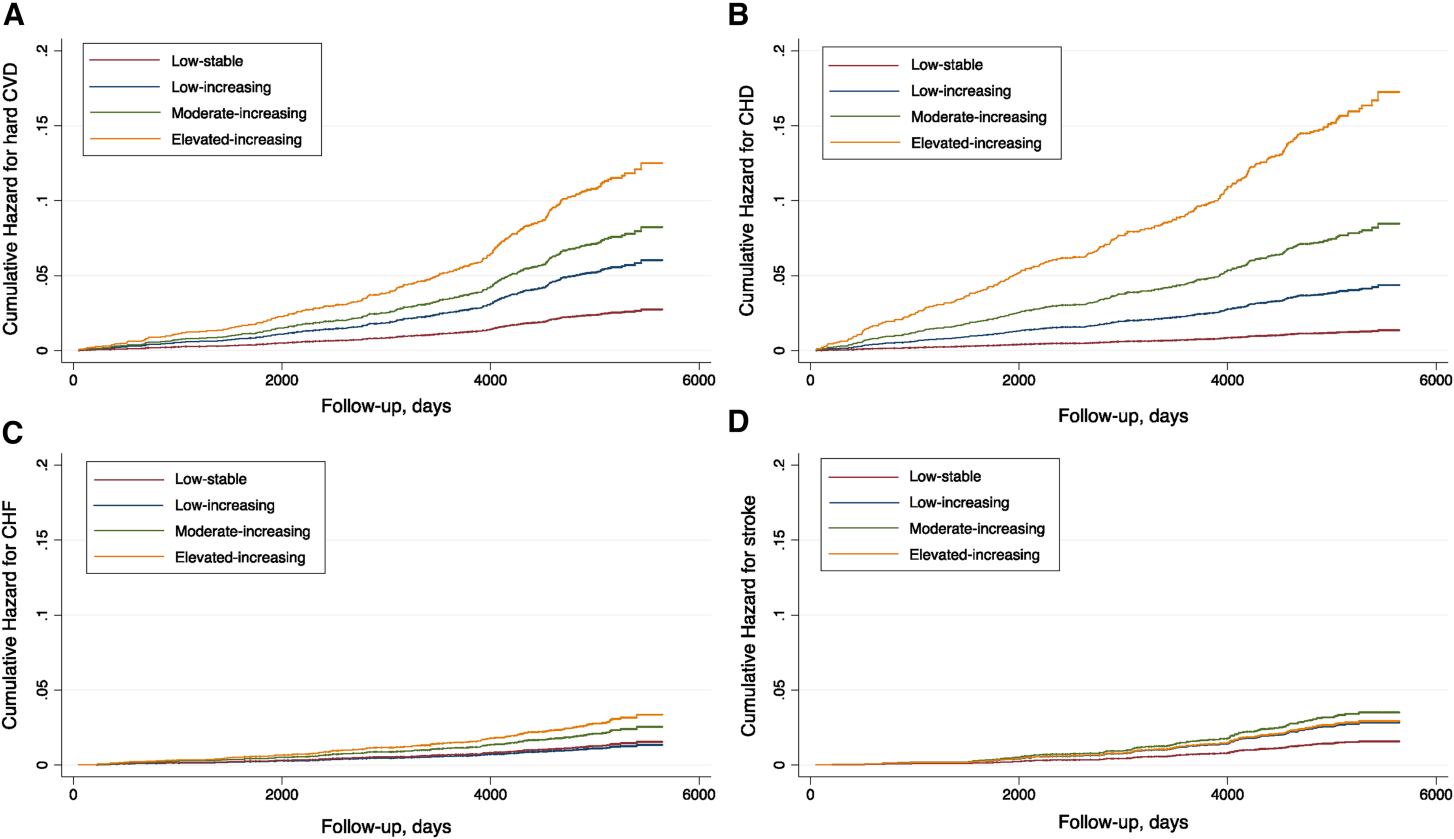
Cumulative hazard plots for the studied outcomes by coronary artery calcium trajectories in the MESA population. (**A**) Hard CVD. (**B**) CHD. (**C**) CHF. (**D**) Stroke.

Similarly, a monotonically increased hazard risk for CHD was found for participants assigned to classes 2, 3, and 4. The full-adjusted HRs were 2.91 (95% CI 1.59–5.31), 5.38 (95% CI 3.06–9.46), and 11.80 (95% CI 6.68–20.87), respectively. With regard to CHF and stroke, the associations were weaker. The increased hazard for CHF in the full-adjusted model was only significantly observed in the elevated-increasing class (class 4), while for stroke, the highest hazard and event rate were noted in the moderate-increasing category. Details of the event rates and hazards adjusted for different covariates are summarized in Table 2. The cumulative hazards for the studied outcomes across different trajectories are plotted in Figure 3.

**Table 2:** HR and 95% CI of the cardiovascular outcomes with coronary artery calcium trajectories.

Outcome	Low-stable	Low-increasing	Moderate-increasing	Elevated-increasing
Cardiovascular events				
Cases/person-years at risk	27/12,128	63/13,688	114/14,761	87/8,389
Event rate (95% CI), per 1,000 person-years	2.23 (1.53–3.25)	4.60 (3.60–5.89)	7.67 (6.38–9.21)	10.37 (8.41–12.80)
Non-adjusted model	Reference	2.08 (1.32–3.26)	3.48 (2.29–5.29)	4.74 (3.07–7.29)
Simple-adjusted model	Reference	2.19 (1.39–3.44)	3.42 (2.24–5.23)	4.97 (3.19–7.75)
Full-adjusted model	Reference	2.10 (1.33–3.01)	3.17 (2.07–4.87)	4.30 (2.73–6.78)
Coronary heart disease				
Cases/person-years at risk	14/12,199	46/13,669	104/14,754	129/7,998
Event rate (95% CI), per 1,000 person-years	1.15 (0.68–1.94)	3.37 (2.52–4.49)	7.05 (5.82–8.54)	16.13 (13.57–19.17)
Non-adjusted model	Reference	2.94 (1.61–5.34)	6.16 (3.53–10.77)	14.15 (8.15–24.56)
Simple-adjusted model	Reference	3.03 (1.67–5.52)	5.86 (3.34–10.28)	14.06 (8.02–24.65)
Full-adjusted model	Reference	2.91 (1.59–5.31)	5.38 (3.06–9.46)	11.80 (6.68–20.87)
Chronic heart failure				
Cases/person-years at risk	19/12,143	21/13,846	49/15,123	44/8,654
Event rate (95% CI), per 1,000 person-years	1.56 (1.00–2.45)	1.52 (0.99–2.33)	3.24 (2.45–4.29)	5.08 (3.78–6.83)
Non-adjusted model	Reference	0.97 (0.52–1.80)	2.08 (1.22–3.53)	3.27 (1.91–5.61)
Simple-adjusted model	Reference	1.00 (0.53–1.86)	2.03 (1.18–3.47)	3.55 (2.03–6.21)
Full-adjusted model	Reference	0.88 (0.47–1.64)	1.72 (0.99–2.97)	2.43 (1.36–4.33)
Stroke				
Cases/person-years at risk	17/12,166	35/13,826	59/15,104	24/8,744
Event rate (95% CI), per 1,000 person-years	1.40 (0.87–2.25)	2.53 (1.82–3.53)	3.90 (3.02–5.04)	2.74 (1.84–4.09)
Non-adjusted model	Reference	1.82 (1.02–3.25)	2.82 (1.65–4.84)	1.99 (1.07–3.70)
Simple-adjusted model	Reference	1.95 (1.09–3.48)	2.88 (1.67–4.98)	2.23 (1.18–4.22)
Full-adjusted model	Reference	1.86 (1.03–3.34)	2.61 (1.50–4.54)	1.87 (0.97–3.61)

Simple-adjusted model: model adjusted for age, gender, race, alcoholic use, education level, marital status, and family income. Full-adjusted model: model adjusted for age, gender, race, alcoholic use, education level, marital status, family income, body mass index, history of hypertension, diabetes, smoking status, physical activity, total energy consumption, dietary calcium, vitamin D and phosphate, LDL-C, HDL-C, total cholesterol, triglycerides, glucose, creatinine, systolic blood pressure, diastolic blood pressure, anti-hypertensive medication, anti-diabetic medication, and lipid-lowering medication.

### Potential risk factors for distinctive CAC trajectories

**Figure 4:**
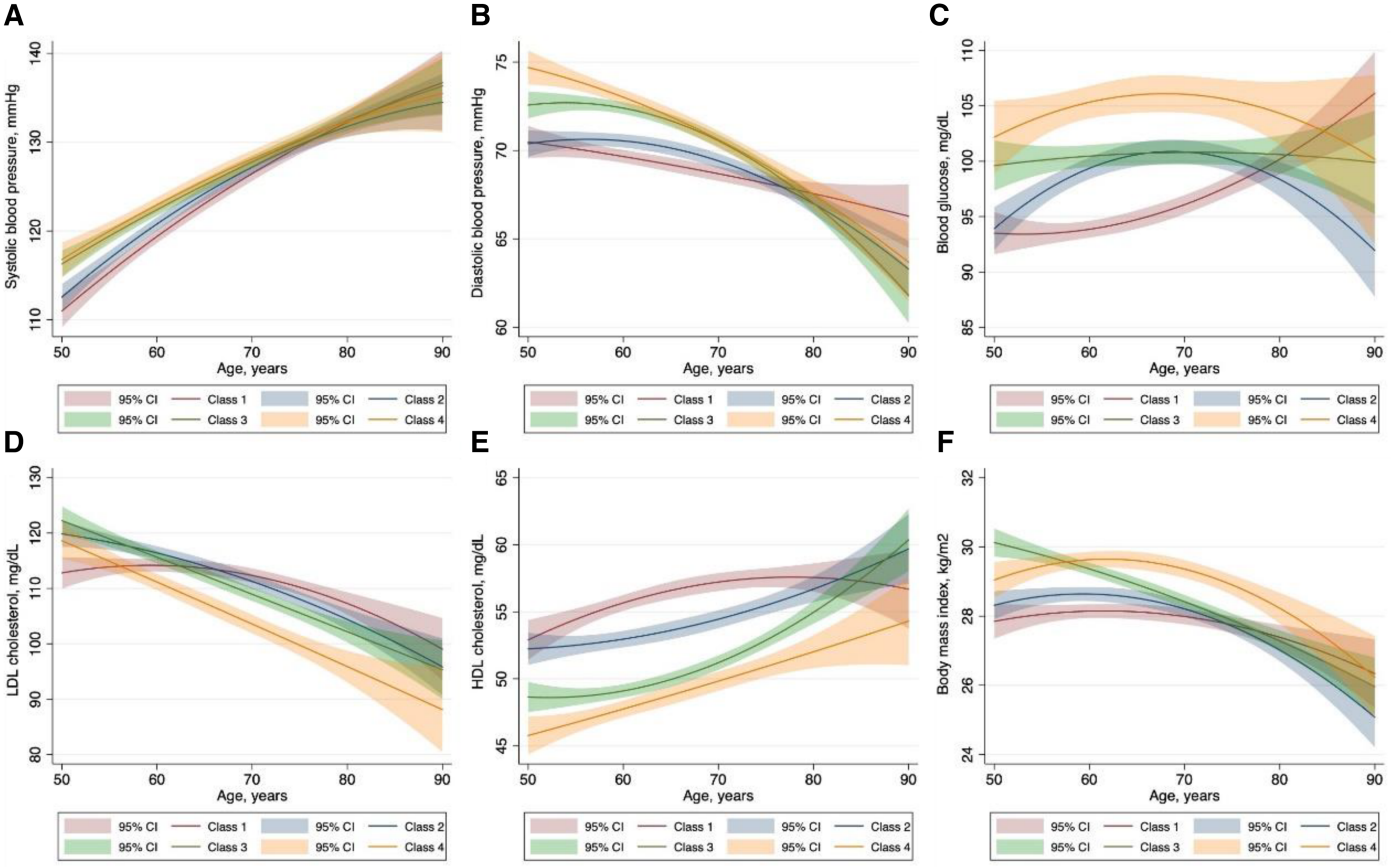
Characteristics of traditional cardiovascular risk factors within each coronary artery calcium trajectory group. The solid lines represent the dynamic change of the variables of interest. The corresponding shaded area represents 95% confidence intervals. Class I: low-stable group; class 2: low-increasing group; class 3, moderate-increasing group; class 4: elevated-increasing group.

To identify the potential risk factors for the observed trajectories, we first examined the available sociodemographic factors in the univariate multinomial logistic regression model (Supplementary Table S3) and further tested significant ones in the multivariate model. As described in Table 3, younger male individuals, with a history of hypertension or diabetes, with lower HDL-C levels had a higher risk of being in the low-increasing category, while smoking, Caucasian race, and higher BMI and LDL-C level further predisposed participants to the moderate-increasing and elevated-increasing categories. We next characterized the dynamic change patterns of traditional cardiovascular risk factors within different CAC trajectories. Overall, patients in the moderate- and elevated-increasing trajectories had higher systolic and diastolic blood pressure, higher fasting glucose, lower HDL-C levels, and higher BMI. However, unexpectedly, participants in the elevated-increasing trajectory had lower LDL-C levels than those in the other trajectory groups (Figure 4).

**Table 3:** Odd ratios (OR) and 95% CI for falling into the identified coronary artery calcium trajectories with potential risk factors using multivariate multinomial logistic regression model.

Risk factors	Low-increasing		Moderate-increasing		Elevated-increasing	
	OR (95%CI)	*p*-value	OR (95%CI)	*p*-value	OR (95%CI)	*p*-value
Age, years	0.97 (0.96–0.98)	<0.001	0.98 (0.97–1.00)	0.008	0.96 (0.95–0.98)	<0.001
Race, Caucasian	1.19 (0.96–1.48)	0.11	1.70 (1.37–2.11)	<0.001	2.79 (2.17–3.58)	<0.001
Gender, male	1.37 (1.07–1.76)	0.01	2.51 (1.96–3.22)	<0.001	5.35 (3.98–7.21)	<0.001
Body mass index, kg/m^2^	0.99 (0.97–1.01)	0.35	1.02 (1.01–1.04)	0.01	1.04 (1.02–1.07)	<0.001
History of hypertension	1.28 (1.04–1.58)	0.02	1.46 (1.19–1.80)	<0.001	2.11 (1.66–2.70)	<0.001
History of diabetes	1.42 (1.11–1.81)	0.005	1.35 (1.06–1.71)	0.02	1.85 (1.41–2.43)	<0.001
Alcoholic use	1.23 (0.96–1.58)	0.10	1.19 (0.92–1.53)	0.36	1.04 (0.75–1.44)	0.80
Education level	1.03 (0.98–1.08)	0.22	1.02 (0.98–1.07)	0.32	1.03 (0.97–1.09)	0.39
Family income	0.97 (0.94–1.00)	0.07	0.96 (0.93–1.00)	0.03	0.97 (0.93–1.02)	0.22
Smoking status	1.05 (0.91–1.21)	0.52	1.16 (1.00–1.34)	0.04	1.44 (1.22–1.70)	<0.001
Creatinine, mg/dl	0.97 (0.59–1.61)	0.92	0.63 (0.38–1.05)	0.07	0.66 (0.36–1.21)	0.18
LDL cholesterol, mg/dl	1.00 (1.00–1.01)	0.07	1.01 (1.00–1.01)	<0.001	1.01 (1.00–1.01)	<0.001
HDL cholesterol, mg/dl	0.99 (0.98–1.00)	0.04	0.98 (0.97–0.99)	<0.001	0.99 (0.98–1.00)	0.004

Being in the low-stable group was taken as the reference outcome.

## Discussion

This is the first study to evaluate the coronary artery calcium trajectory over a long period. In the present analysis, we identified four unique trajectories in CAC over a 10-year span. Participants falling into the low-increasing, moderate-increasing, and elevated-increasing trajectories experienced significantly increased hazards for hard CVD and total CHD when compared to participants in the low-stable group. The graded positive associations with hard CVD were consistently observed in subgroups of age, sex, and race, with the presence or absence of hypertension or diabetes. Interestingly, risk factors for falling into the distinct trajectories are not equally the same, suggesting that the driven cause for coronary artery calcification and the accelerating factors may differ.

Recently, authors from the Heinz Nixdorf Recall (HNR) study developed a mathematical tool for predicting CAC progression and proposed that CAC progression seemed to be inevitable once CAC level exceeds 10 (8). This view is supported by the results in our study. Over the 10-year span, coronary calcium burden generally increased, rising from a median baseline CAC score of 20 in the low-increasing trajectory. Although various methods are used to define CAC progression, its predictive value on cardiovascular risk has not yet been confirmed. Most of the previous publications suggest a positive effect of progression rate on cardiovascular risks (10, 11, 15). However, in a more recent analysis of the HNR study, CAC progression adds only weakly to risk prediction (13).

Our study extends and complements the previous findings. As depicted in Figure 1, despite the similar rate of progression, participants in the moderate-increasing trajectory had higher risks than those in the low-increasing trajectory. Likewise, although a slower progression rate was observed in the elevated-increasing category, it still portended the highest hazard for CVD. This further supports the limitation of simply using progression rate when assessing the cardiovascular risk. In particular, the overall baseline level should also be emphasized. Usually, investigators tend to categorize subjects into subgroups of 0, 0–100,100–400, and 400+, representing increasing degrees of severity (2, 3). However, in this study, we found a lower median baseline calcium score for each of the progressing trajectories of 0, 20, and 250, respectively. This raises a question on whether a lower cutoff should be used to define the risk categories.

Most traditional cardiovascular risk factors, including age, male sex, smoking status, hypertension, obesity, diabetes mellitus, and family history of heart attack, have been reported to be associated with incident CAC and CAC progression. However, owing to inconsistent definitions used in different studies, the predictors of CAC progression also varied widely. For instance, in the study conducted by Koulaouzidis et al., history of smoking, diabetes, or hypertension was found to be significantly associated with the risk of incident CAC (28), while in another study, incident conversion to a positive CAC score was significantly related only to diabetes and smoking (29).

Pathophysiologically, stimuli in the initiation and progression of calcification may vary on the basis of the plaque status and surroundings (5). Therefore, the risk factors for the onset and progression of CAC could possibly differ. In general, in our study, age, male sex, and history of hypertension and diabetes are consistently associated with the low-, moderate-, and elevated-increasing trajectories. However, certain factors appeared to be related only to risk of progression but not to incident CAC (i.e., Caucasian race, cigarette smoking, and a higher BMI). This disparity may reveal additional insight into the pathogenesis of CAC development or progression. Notably, although CAC is more prevalent in older individuals, the risk for progression tends to occur at a younger age. This is in part in accordance with findings from the HNR study, in which younger men were more prone to CAC progression than elderly participants (8). That is, exposure to some risk factors may initiate the conversion to a positive CAC, while persistent elevation of part of these factors further promotes their lifetime accumulation. That suggested clinics should advise different risk factor management strategies for patients according to their potential CAC trajectories. In this regard, our findings could be viewed as important progress for disease prevention. On the other hand, longitudinally plotting the characteristics of traditional cardiovascular risk factors within different CAC trajectories further enlightens our understanding of the effect of specific risk factors on CAC progression.

The key strength of our study is availability of multiple repetitive measurements of CAC during a long follow-up duration and using GBTM, which entails classifying heterogeneous individuals into homogeneous groups. Understanding the characteristics of participants who follow different trajectory patterns has the potential to inform how to intervene at an earlier stage and instruct future development of individualized risk management. However, whether a more intense risk modification strategy could alter an individual's long-term patterns of CAC change and prevent future cardiac events warrants further study.

Despite the abovementioned strengths, the present study has some limitations. First, although the trajectories were chosen based on high AVPP and low BIC, participants could still be assigned into a misclassified group. Second, although repeated CT measurements were performed per protocol, selection bias could still exist. Scores of zero on the initial scan portend a lower likelihood of performing a repeated scanning, thereby underestimating the proportion of the low-stable trajectory population and overestimating the corresponding increasing trajectories. Finally, participants in our study are mainly a population of middle-aged to older individuals free of CVD at baseline. This limits the generalizability to other age groups and individuals with pre-existing CVD.

## Conclusions

For the first time we identified four unique trajectories in CAC change over a long period. In particular, participants falling into the low-increasing, moderate-increasing, and elevated-increasing trajectories experienced significantly increased risks for hard CVD.

## Data Availability

The original contributions presented in the study are included in the article/**Supplementary Material**, further inquiries can be directed to the corresponding author.
