# Supplementary material for: Ten-year trajectory of coronary artery calcification and risk of cardiovascular outcomes: the Multi-Ethnic Study of Atherosclerosis
Source: Front Cardiovasc Med. 2024 Jul 10;11:1406216. doi: 10.3389/fcvm.2024.1406216 (PMC11266147; doi:10.3389/fcvm.2024.1406216)
Supplement: Supplementary file 1 [file Datasheet1.docx]

**eMethods**

Initially, for each model, the linear, quadratic and cubic functions of each trajectory can be tested, depending on the number of time points. As a general rule, for data sets with 3 time points, a single quadratic trajectory model is tested first. Next, more complex models with an increasing number of trajectories were continued testing in order to determine which model provides the best fit to the data.

This process of comparing the fit of each subsequent, more complex model, to the fit of the previously tested, simpler model, continues until there is no substantial evidence for improvement in model fit. The fit of each model is compared using the estimate of the log Bayes factor (1). The estimate is approximately equal to two times the difference in the Bayesian information criterion (BIC) values for the two models being compared: 2*(BIC _More complex model_-(BIC _simpler model_). According to the suggested guidelines, values greater than 10 are interpreted as very strong evidence of better fit (2). In addition, the averaged posterior probabilities for each trajectory are examined to evaluate the tenability of each model. The adequacy of the final model was evaluated on the basis of a low Bayesian information criterion (BIC) and higher than 0.70 probability of belonging (2,3).

In this analysis, we first modelled the data with one quadratic trajectory (model 1). In each output, statistics are provided for each estimated parameter including the intercept (β_0_) , the linear parameter (β_1_) , and the quadratic parameter (β_2_) for each trajectory.

The results of model 1 can be summarized as follows: β_0_ = -22.33, P <0.001; β_1_=0.53, P <0.001; and β_2_ = -0.003, P <0.001; BIC = -21554 (model 1).

Since the quadratic component of the above model is significant, analysis for the quadratic model for two trajectories is performed (model 2). The output is presented below:

Trajectory 1: β_0_ = -18.81, P <0.001; β_1_=0.34, P <0.001; and β_2_ = -0.001, P =0.04;

Trajectory 2: β_0_ = -18.50, P <0.001; β_1_=0.58, P <0.001; and β_2_ = -0.003, P <0.001;

BIC = -17971 (model 2).

log Bayes factor=2*[(-17971) - ( -21554)] =7166, which indicates that the quadratic model for two trajectories (model 2) is fitting better than one trajectory (model 1).

This process is repeated with an increasing number of trajectories until the model of best fit is obtained. The results of analysis for the quadratic model for three trajectories are presented (model 3).

Trajectory 1: β_0_ = 0.05, P =1; β_1_=1.94, P =0.99; and β_2_ = -0.04, P =0.99;

Trajectory 2: β_0_ = -23.09, P <0.001; β_1_=0.51, P <0.001; and β_2_ = -0.002, P <0.001;

Trajectory 3: β_0_ = -14.98, P <0.001; β_1_=0.49, P <0.001; and β_2_ = -0.002, P <0.001;

BIC = -17513 (model 3).

To ensure parsimony, non‐significant quadratic terms are removed but linear parameters are retained from trajectories in a given model. The updated model is then retested yielding a new BIC value.

model 4:

Trajectory 1: β_0_ = -16.70, P <0.001; β_1_=0.22, P <0.001;

Trajectory 2: β_0_ = -32.82, P <0.001; β_1_=0.86, P <0.001; and β_2_ = -0.005, P <0.001;

Trajectory 3: β_0_ = -13.32, P <0.001; β_1_=0.47, P <0.001; and β_2_ = -0.003, P <0.001;

BIC = -16925 (model 4).

model 4 vs model 2:

log Bayes factor=2*[(-16925) - ( -17971)] = 2092, indicating model 4 is a better fit than model 2.

model 5:

Trajectory 1: β_0_ = -17.53, P <0.001; β_1_=0.27, P <0.001;

Trajectory 2: β_0_ = -99.40, P <0.001; β_1_=0.22, P <0.001; and β_2_ = -0.01, P <0.001;

Trajectory 3: β_0_ = -33.82, P <0.001; β_1_=0.95, P <0.001; and β_2_ = -0.006, P <0.001;

Trajectory 4: β_0_ = -11.79, P <0.001; β_1_=0.45, P <0.001; and β_2_ = -0.003, P <0.001;

BIC = -16228 (model 5).

model 5 vs model 4

log Bayes factor=2*[(-16228)- (-16925)] =1394>10, indicating model 5 is a better fit than model 4.

model 6:

Trajectory 1: β_0_ = -22.27, P <0.001; β_1_=0.28, P <0.001;

Trajectory 2: β_0_ = -33.74, P <0.001; β_1_=0.97, P <0.001; and β_2_ = -0.006, P <0.001;

Trajectory 3: β_0_ = -84.80, P <0.001; β_1_=2.01, P <0.001; and β_2_ = -0.01, P <0.001;

Trajectory 4: β_0_ = -54.58, P <0.001; β_1_=1.41, P <0.001; and β_2_ = -0.008, P <0.001;

Trajectory 5: β_0_ = -12.72, P <0.001; β_1_=0.49, P <0.001; and β_2_ = -0.003, P <0.001;

BIC = -15711 (model 6).

model 6 vs model 5

log Bayes factor=2*[( -15711)- (-16228)] =1034, indicating model 6 is a better fit than model 5.

model 7:

Trajectory 1: β_0_ = -22.27, P <0.001; β_1_=0.28, P <0.001;

Trajectory 2: β_0_ = -33.74, P <0.001; β_1_=0.97, P <0.001; and β_2_ = -0.006, P <0.001;

Trajectory 3: β_0_ = -84.80, P <0.001; β_1_=2.01, P <0.001; and β_2_ = -0.01, P <0.001;

Trajectory 4: β_0_ = -54.58, P <0.001; β_1_=1.41, P <0.001; and β_2_ = -0.008, P <0.001;

Trajectory 5: β_0_ = -12.72, P <0.001; β_1_=0.49, P <0.001; and β_2_ = -0.003, P <0.001;

Trajectory 6: β_0_ = -12.72, P <0.001; β_1_=0.49, P <0.001; and β_2_ = -0.003, P <0.001;

BIC = -15598 (model 7).

model 7 vs model 6

log Bayes factor=2*[ (-15598) - (-15711)] =226, indicating model 7 is a better fit than model 6.

Next, the averaged posterior probabilities (AVPP) of trajectories within each model are calculated for the above models. The evaluation indexes are summarized in the below table.

|  | BIC | traj1 | traj 2 | traj 3 | traj 4 | traj 5 | traj 6 |
| --- | --- | --- | --- | --- | --- | --- | --- |
| model 1 | -21554 | 1 | - | - | - | - | - |
| model 2 | -17971 | 0.98 | 0.97 | - | - | - | - |
| model 3 | -17513 | 0.81 | 0.84 | 0.96 | - | - | - |
| model 4 | -16925 | 0.92 | 0.9 | 0.91 | - | - | - |
| *model 5* | *-16228* | *0.82* | *0.78* | *0.88* | *0.90* | *-* | *-* |
| model 6 | -15711 | 0.84 | 0.86 | **0.65** | 0.77 | 0.89 | - |
| model 7 | -15598 | 0.73 | 0.84 | **0.65** | 0.74 | 0.82 | 0.84 |

On the basis of both a low BIC value and higher than 0.7 probabilities of belong, the model with three quadratic trajectories and one linear trajectory (model 5) is retained as the final and most parsimonious model in our study.

***Reference***

1.Andruff H, Carraro N, Thompson A, Gaudreau P. Latent Class Growth Modeling: A Tutorial. Tutorials in Quantitative Methods for Psychology. 2009;5(1):11-24

2.Jones, B.L., Nagin, D.S., & Roeder, K. A SAS procedure based on mixture models for estimating developmental trajectories. Sociological Methods & Research. 2001;29:374‐393.

3.Besen E, Pransky G. Trajectories of productivity loss over a 20-year period: an analysis of the National Longitudinal Survey of Youth. Scandinavian Journal of Work, Environment & Health. 2014;40(4):380-9.

4.Jung T, Wickrama KAS. An Introduction to Latent Class Growth Analysis and Growth Mixture Modeling. Social and Personality Psychology Compass. 2008;2(1):302-17.

eTable 1 Proportion of missing values in the main analysis.

|  | Complete | Incomplete | Imputed | Total | Missing, % |
| --- | --- | --- | --- | --- | --- |
| Alcoholic use | 3612 | 4 | 4 | 3616 | 0.11 |
| Educational level | 3612 | 4 | 4 | 3616 | 0.11 |
| Marital status | 3612 | 4 | 4 | 3616 | 0.11 |
| Family income | 3507 | 109 | 109 | 3616 | 3.01 |
| History of diabetes | 3607 | 9 | 9 | 3616 | 0.25 |
| LDL cholesterol, mg/dl | 3559 | 57 | 57 | 3616 | 1.58 |
| Glucose, mg/dl | 3607 | 9 | 9 | 3616 | 0.25 |
| HDL cholesterol, mg/dl | 3603 | 13 | 13 | 3616 | 0.36 |
| Creatinine, mg/dl | 3607 | 9 | 9 | 3616 | 0.25 |
| Smoking status | 3612 | 4 | 4 | 3616 | 0.11 |
| Physical activity, MET-min/week | 3613 | 3 | 3 | 3616 | 0.08 |
| Systolic BP, mmHg | 3431 | 185 | 185 | 3616 | 5.12 |
| Diastolic BP, mmHg | 3431 | 185 | 185 | 3616 | 5.12 |
| Anti-hypertensive medication | 3615 | 1 | 1 | 3616 | 0.03 |
| Lipid-lowering medications | 3615 | 1 | 1 | 3616 | 0.03 |
| Anti-diabetic medication | 3613 | 3 | 3 | 3616 | 0.08 |
| Dietary vitamin D, mcg | 3316 | 300 | 300 | 3616 | 8.30 |
| Dietary calcium, mg | 3316 | 300 | 300 | 3616 | 8.30 |
| Dietary energy, kcal | 3316 | 300 | 300 | 3616 | 8.30 |
| Dietary phosphate, mg | 3316 | 300 | 300 | 3616 | 8.30 |

Abbreviation: LDL, low-density lipoprotein; HDL, high-density lipoprotein; BP, blood pressure.

eTable 2 Hazard ratio (HR) and 95% confidence intervals (CI) of hard cardiovascular disease (CVD) with CAC trajectories in subgroup and sensitivity analysis.

|  | Low-stable | Low-increasing | Moderate-increasing | Elevated-increasing |
| --- | --- | --- | --- | --- |
| Age, years |  |  |  |  |
| <=65 | reference | 1.59(0.74,3.41) | 2.44(1.19,4.98) | 4.33(2.08,9.01) |
| >65 | reference | 2.47(1.23,4.97) | 3.57(1.82,7.00) | 4.46(2.11,9.42) |
| Gender |  |  |  |  |
| Male | reference | 2.85(1.16,7.01) | 4.51(1.92,10.60) | 6.37(2.67,15.20) |
| Female | reference | 1.94(1.03,3.65) | 2.28(1.21,4.30) | 3.70(1.79,7.65) |
| Race |  |  |  |  |
| Caucasian | reference | 2.49(1.05,5.93) | 3.62(1.57,8.34) | 3.43(1.42,8.25) |
| Non-Caucasian | reference | 2.19(1.16,4.13) | 2.84(1.54,5.21) | 6.08(3.22,11.46) |
| Hypertension | |  |  |  |
| Yes | reference | 2.26(1.13,4.53) | 2.90(1.48,5.67) | 4.69(2.34,9.41) |
| No | reference | 2.31(1.08,4.90) | 3.36(1.64,6.88) | 4.41(2.01,9.66) |
| Diabetes |  |  |  |  |
| Yes | reference | 1.94(0.67,5.68) | 3.97(1.47,10.76) | 6.29(2.26,17.50) |
| No | reference | 2.42(1.35,4.33) | 2.90(1.65,5.12) | 4.13(2.24,7.60) |
| Excluding participants with  missing values at baseline | reference | 2.20(1.32,3.65) | 3.00(1.84,4.88) | 4.55(2.72,7.62) |

Model was adjusted for age, gender, race, alcoholic use, education level, marital status, family income, body mass index, history of hypertension, diabetes, smoking status, physical activity, total energy consumption, dietary calcium, vitamin D and phosphate. LDL-C, HDL-C, total cholesterol, triglycerides, glucose, creatinine, systolic blood pressure, diastolic blood pressure, anti-hypertensive medication, anti-diabetic medication, lipid-lowering medication. 479 participate were excluded due to missing data.

eTable 3 Potential risk factors for the identified trajectories using univariate multinomial logit regression model.

| Potential risk factors | Low-increasing | | Moderate-increasing | | Elevated-increasing | |
| --- | --- | --- | --- | --- | --- | --- |
|  | OR(95%CI) | P value | OR(95%CI) | P value | OR(95%CI) | P value |
| Age, years | 0.98(0.97,0.99) | <0.001 | 0.99(0.98,1.01) | 0.09 | 0.98(0.97,0.99) | <0.001 |
| Race, Caucasian | 1.08(0.89,1.31) | 0.44 | 1.42(1.18,1.71) | <0.001 | 2.10(1.70,2.59) | <0.001 |
| Gender, male | 1.51(1.24,1.82) | <0.001 | 2.64(2.20,3.18) | <0.001 | 5.42(4.34,6.77) | <0.001 |
| Body mass index, kg/m2 | 1.00(0.99,1.02) | 0.60 | 1.03(1.02,1.05) | <0.001 | 1.05(1.03,1.07) | <0.001 |
| History of hypertension | 1.05(0.88,1.27) | 0.58 | 1.26(1.05,1.51) | 0.01 | 1.57(1.28,1.93) | <0.001 |
| History of diabetes | 1.35(1.08,1.69) | 0.009 | 1.55(1.25,1.93) | <0.001 | 2.04(1.61,2.60) | <0.001 |
| Alcoholic use | 1.33(1.07,1.65) | 0.01 | 1.67(1.34,2.08) | <0.001 | 2.28(1.72,3.02) | <0.001 |
| Education level | 1.03(0.99,1.07) | 0.19 | 1.02(0.99,1.06) | 0.24 | 1.07(1.02,1.11) | 0.004 |
| Marital status | 0.98(0.92,1.05) | 0.56 | 1.00(0.94,1.07) | 0.98 | 0.96(0.89,1.03) | 0.27 |
| Family income | 1.01(0.98,1.04) | 0.51 | 1.01(0.99,1.04) | 0.38 | 1.07(1.03,1.10) | <0.001 |
| Smoking status | 1.14(0.99,1.31) | 0.06 | 1.33(1.16,1.51) | <0.001 | 1.69(1.46,1.97) | <0.001 |
| Systolic BP, mmHg | 1.00(0.99,1.01) | 0.87 | 1.01(1.00,1.01) | 0.006 | 1.01(1.01,1.02) | <0.001 |
| Diastolic BP, mmHg | 1.01(0.99,1.01) | 0.26 | 1.02(1.01,1.03) | <0.001 | 1.04(1.03,1.05) | <0.001 |
| Creatinine, mg/dl | 1.50(0.93,2.40) | 0.10 | 2.59(1.65,4.08) | <0.001 | 4.96(3.03,8.11) | <0.001 |
| Glucose, mg/dl | 1.01(1.00,1.01) | 0.008 | 1.01(1.01,1.02) | <0.001 | 1.02(1.01,1.02) | <0.001 |
| LDL cholesterol, mg/dl | 1.00(1.00,1.01) | 0.08 | 1.00(1.00,1.01) | 0.002 | 1.01(1.00,1.01) | 0.001 |
| HDL cholesterol, mg/dl | 0.99(0.98,0.99) | <0.001 | 0.97(0.96,0.98) | <0.001 | 0.96(0.95,0.97) | <0.001 |
| Total cholesterol, mg/dl | 1.00(1.00,1.00) | 0.78 | 1.00(1.00,1.00) | 0.74 | 1.00(1.00,1.00) | 0.37 |
| Triglycerides, mg/dl | 1.00(1.00,1.00) | 0.76 | 1.00(1.00,1.00) | <0.001 | 1.00(1.00,1.00) | <0.001 |
| Dietary vitamin D, mcg | 0.99(0.97,1.02) | 0.73 | 1.00(0.98,1.03) | 0.89 | 1.01(0.99,1.04) | 0.34 |
| Dietary calcium, mg | 1.00(1.00,1.00) | 0.75 | 1.00(1.00,1.00) | 0.97 | 1.00(1.00,1.00) | 0.53 |
| Dietary phosphate, mg | 1.00(1.00,1.00) | 0.48 | 1.00(1.00,1.00) | 0.29 | 1.00(1.00,1.00) | 0.008 |
| Physical activity, MET-min/week | 1.00(1.00,1.00) | 0.04 | 1.00(1.00,1.00) | 0.03 | 1.00(1.00,1.00) | <0.001 |

Being in the low-stable group was taken as the reference outcome. OR, odds ratio; CI, confidence intervals.
